# Unique Features of *Entamoeba* Sulfur Metabolism; Compartmentalization, Physiological Roles of Terminal Products, Evolution and Pharmaceutical Exploitation

**DOI:** 10.3390/ijms20194679

**Published:** 2019-09-21

**Authors:** Fumika Mi-ichi, Hiroki Yoshida

**Affiliations:** Division of Molecular and Cellular Immunoscience, Department of Biomolecular Sciences, Faculty of Medicine, Saga University, 5-1-1 Nabeshima, Saga 849-8501, Japan; yoshidah@med.saga-u.ac.jp

**Keywords:** amoebiasis, mitosome, lipid metabolism, encystation, lateral gene transfer

## Abstract

Sulfur metabolism is essential for all living organisms. Recently, unique features of the *Entamoeba* metabolic pathway for sulfated biomolecules have been described. *Entamoeba* is a genus in the phylum Amoebozoa and includes the causative agent for amoebiasis, a global public health problem. This review gives an overview of the general features of the synthesis and degradation of sulfated biomolecules, and then highlights the characteristics that are unique to *Entamoeba*. Future biological and pharmaceutical perspectives are also discussed.

## 1. Introduction

### 1.1. Sulfur Metabolism

Sulfur is an essential element for life in all living organisms. Sulfate in the environment is typically activated as a prerequisite step for both sulfation and sulfate assimilation pathways. Sulfate activation is broadly found throughout the Bacteria, Protista, Fungi, Plantae, and Metazoa kingdoms [1,2]; KEGG (see Box 1).

Box 1 **Databases.**

Sulfur metabolism research has greatly benefited from ever-expanding databases, such as the Kyoto Encyclopedia of Genes and Genomes (KEGG) (https://www.genome.jp/kegg/), The National Center for Biotechnology Information (NCBI) (https://www.ncbi.nlm.nih.gov/), and AmoebaDB (https://amoebadb.org/amoeba/). The information available in such databases is, therefore, cited in this review where necessary.

**Figure 1:**
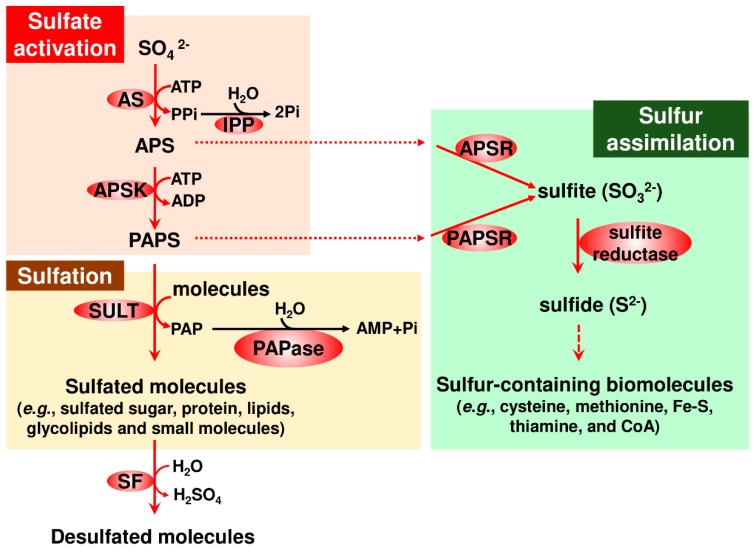
Overview of sulfate metabolism functionally linked to sulfate activation. An overall metabolic pathway is depicted, in which information from bacterial to human studies is integrated. Note that *Entamoeba* does not possess genes encoding APS and PAPS reductases (AmoebaDB; KEGG). The enzyme responsible for each step is shown by a filled oval. Abbreviations used: APS, adenosine 5′-phosphosulfate; APSK, APS kinase; AS, ATP sulfurylase; IPP, inorganic pyrophosphatase; PAP, adenosine 3′,5′-bisphosphate; PAPase, 3′(2′),5′-bisphosphate nucleotidase; PAPS, 3′-phosphoadenosine 5′-phosphosulfate; Pi, inorganic phosphate; PPi, inorganic pyrophosphate; SF, sulfatase; and SULT, sulfotransferase. Enzymatic reactions are indicated by arrows. Multiple steps for synthesizing sulfur-containing biomolecules is simplified by dashed arrow. Fine dotted lines indicate molecular transfer of APS and PAPS.

Sulfate taken up from the external milieu is activated by two sequential reactions to provide adenosine 5′-phosphosulfate (APS) and 3′-phosphoadenosine 5′-phosphosulfate (PAPS), respectively. PAPS then acts as a sulfate donor in a sulfotransferase (SULT) reaction, in which sulfate is transferred to a variety of acceptors, to generate sulfated sugars, proteins, lipids, glycolipids and small organic molecules [3,4]. The various products generated are often hydrolyzed by sulfatase (SF) [4,5]. Meanwhile, some organisms, such as plants, APS and PAPS, serve as intermediates in branched pathways and in sulfate assimilation pathways for synthesizing a wide array of sulfur-containing biomolecules crucial for living organisms, such as thiol-containing amino acids (cysteine and methionine) and sulfur-containing cofactors (Fe-S clusters, thiamine, and coenzyme A (CoA)) [2,3,4]. An overview of the metabolic pathways that are functionally linked to sulfate activation is illustrated in Figure 1.

### 1.2. Entamoeba Histolytica Infection Causes Amoebiasis

**Figure 2:**
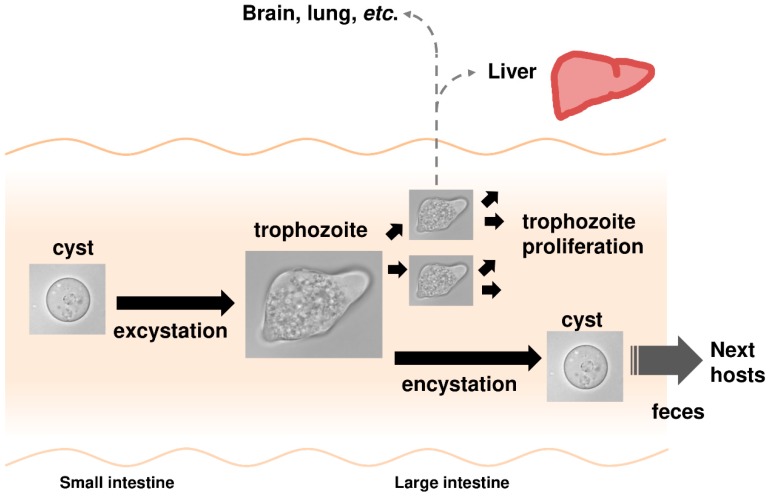
*Entamoeba histolytica*, the causative agent for amoebiasis. Schematic illustration of the main route of infection and behavior inside the human host is shown. The life cycle of *E*. *histolytica* essentially consists of proliferative trophozoite and dormant cyst stages.

*E*. *histolytica* belongs to the phylum Amoebozoa in the kingdom Protista. *E*. *histolytica* infection causes amoebiasis, a parasitic disease that is prevalent worldwide [6,7,8,9]. The infection usually occurs by the oral ingestion of cysts. The ingested cysts then travel to the small intestine where they hatch into trophozoites. Trophozoites then descend to the large intestine and proliferate there. Some proliferating trophozoites invade the mucosal tissue and can be transported via the blood stream to other organs apart from the intestine where they can form abscesses, most commonly in the liver. Meanwhile, some trophozoites in the large intestine differentiate into dormant cysts, which are then excreted in feces (Figure 2) [10]. The water or food contaminated by feces from infected individuals is the principal source of amoebiasis [8,9]. Therefore, the parasitic life cycle of *E*. *histolytica* is essentially maintained by alternating its proliferative trophozoite and dormant cyst forms. The proliferative and invasive capabilities of trophozoites cause typical amoebiasis symptoms, such as dysentery and hepatic abscess. Meanwhile, disease transmission requires the dormancy of cysts and the high occurrence of asymptomatic patients who unconsciously spread the disease is a serious public health problem [9]. Current clinical options are inadequate. Available drugs are limited and an effective vaccine has not been developed [6,8]. Hence, new measures for killing trophozoites and halting cyst formation, such as anti-amoebic and amoebiasis transmission-blocking drugs, are urgently needed.

This review focuses on *Entamoeba* sulfur metabolism and highlights its unique features of spatial compartmentalization, the terminal products produced and their roles in physiology and evolution. *Entamoeba* sulfur metabolism from pharmaceutical and biological perspectives is also discussed.

## 2. Biosynthesis and Degradation of Sulfated Biomolecules That Are Crucial for Homeostasis

### 2.1. General Features of Sulfate Activation

Sulfate activation is a highly conserved and pivotal step in the sulfation pathway [4]. This step consists of two sequential reactions mediated by ATP sulfurylase (AS) and APS kinase (APSK) [3,4]. AS reversibly catalyzes adenylyl-transfer from ATP to sulfate to produce APS and inorganic pyrophosphate (PPi). However, the direction toward APS generation is thermodynamically reversible, while APSK irreversibly catalyzes phosphate-transfer from ATP to APS to produce PAPS and ADP. These two enzymes are linked by inorganic pyrophosphatase (IPP), which overcomes the thermodynamic barrier of the AS-catalyzed reaction, resulting in the facilitation of sulfate activation. IPP hydrolyzes PPi to two inorganic phosphates, which lowers the level of PPi generated from the energetically unfavorable AS-catalyzed reaction. Consequently, the AS-catalyzed reaction favors APS production. Overall, a high level of APS promotes sulfate activation toward producing PAPS, a versatile metabolite in sulfation (see Figure 1).

### 2.2. Sulfotransferases (SULTs)

SULT catalyzes sulfate-transfer from PAPS to an acceptor. The genes encoding this enzyme are widely distributed in the genomes of organisms in all three domains, Archaea, Bacteria, and Eukarya ([11]; KEGG; NCBI). However, SULTs have only been studied in a small number of organisms. In humans, large numbers of SULTs have been characterized and they have participated in a variety of processes of medical importance [5,12]. Compared with studies of mammalian SULTs, the investigations of SULTs in other organisms, such as plants and bacteria, are relatively recent [4,13].

A remarkable array of substrates is sulfated by members of the human SULT superfamily. Sulfated substrates include small molecules (cholesterol and catecholamine), steroids (estrogen and dehydroepiandrosterone), peptide hormones (gastrin, cholestocystokinin, and thyroid-stimulating and -luteinizing hormones), glycosaminoglycans (heparan sulfate, chondroitin sulfate, and ketaran sulfate), and proteins (chemokine receptor, P-selectin glycoprotein ligand 1, and sialyl Lewis^x^ glycoprotein). For peptide and protein substrates, hydroxyl groups of certain tyrosine residues are sulfated. The products have a broad array of functions, such as xenobiotic detoxification, cell differentiation and development, and immune response actions [12,14,15]. 

Plant SULTs are similar to mammalian enzymes and also utilize a broad range of substrates as the sulfate acceptor. The sulfated products are suggested to play important roles in homeostasis. The substrates characterized to date are mostly plant hormones and their precursors, which are small molecules and peptides. In some plants, sulfated polysaccharides, which are likely synthesized by putative SULTs, are also found [13].

In contrast to the wide variety of sulfated molecules that have been identified and characterized as products of mammalian and plant SULTs, a relatively limited number of sulfated molecules have been identified in bacteria and are mostly sulfated glycolipids [16]. For instance, *Mycobacterium tuberculosis*, the causative agent for tuberculosis, synthesizes a structurally determined sulfated glycolipid, termed SL-1, and sulfated glycopeptide lipids [17]. Their involvement in virulence was also shown [16,18].

### 2.3. Sulfatases (SFs)

SFs hydrolyze sulfated molecules and also play crucial roles in many organisms [4,5,19,20,21,22]. Human SFs are involved in inherited metabolic diseases, such as multiple SF deficiency, mucopolysaccharoidoses, and metachromatic mukodystrophy [19]. In addition, SFs are involved in diseases, such as breast cancer, gynecological diseases, arteriosclerosis, and angiogenesis [23,24]. Furthermore, the SF family in microbes that colonize the human gut, known as the gut microbiota, has emerged as a key factor in the symbiotic interaction between gut microbiota and the host [21,22]. This interaction plays a crucial role in human physiology and an imbalance in this symbiotic interaction (called dysbiosys) potentially causes metabolic and immune diseases, such as obesity, inflammatory bowel disease, diabetes, hepatic diseases, Crohn’s disease, colorectal cancer, and allergy [21].

SFs are classified into three mechanistically and structurally distinct groups: The alkylSFs from the α-ketoglutarate-dependent dioxygenase family; the Zn-dependent alkylSFs, and the formylglycine-dependent SFs, which are highly prevalent among organisms and are active on a broad range of substrates [19,20,22].

## 3. Unique Features of Sulfated Molecule Metabolism in *Entamoeba*

**Figure 3:**
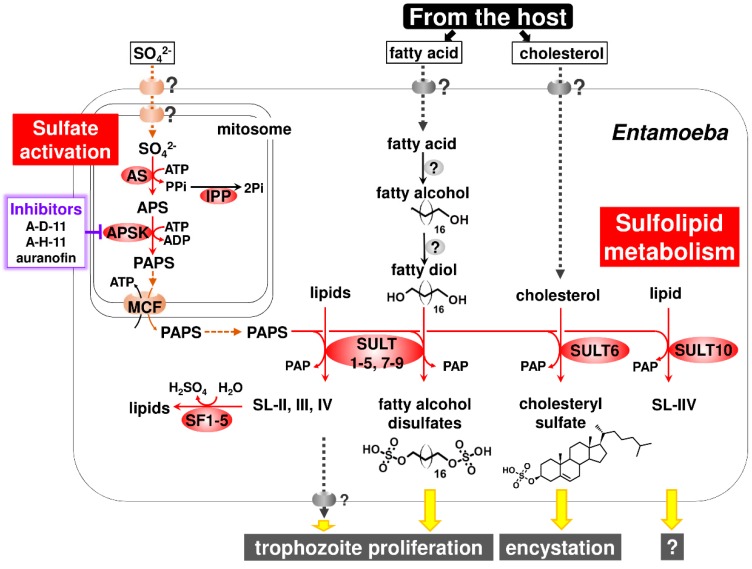
*Entamoeba* sulfolipid metabolism. The entire pathway for the synthesis and degradation of sulfolipids, the pleiotropic roles of sulfolipids, the terminal metabolites for maintenance of the parasitic lifestyle, and rational targets for amoebiasis therapy are shown. Abbreviations used; APS, adenosine 5′-phosphosulfate; APSK, APS kinase; AS, ATP sulfurylase; IPP, inorganic pyrophosphatase; MCF, mitochondrial carrier family; PAP, adenosine 3′,5′-bisphosphate; PAPase, 3′(2′),5′-bisphosphate nucleotidase; PAPS, 3′-phosphoadenosine 5′-phosphosulfate; Pi, inorganic phosphate; PPi, inorganic pyrophosphate; SF, sulfatase; and SULT, sulfotransferase. Predicted roles of sulfolipids in *Entamoeba* life cycle are indicated by yellow arrows. Putative transporters, the presence of which is unknown, are illustrated on the membrane.

This section highlights the uniqueness of *Entamoeba* sulfated molecule synthesis and degradation, in which three enzymes crucial for sulfate activation, AS, APSK, and IPP, and SULT and SF families cooperate in sulfolipid metabolism (Figure 3). These unique features include sulfate activation atypically localized in mitochondria-related organelles (MROs) known as mitosomes in *Entamoeba*, terminal metabolites and their roles in the maintenance of the parasitic lifestyle, and pathways linked to sulfolipid metabolism and evolution.

### 3.1. Atypical Localization of Sulfate Activation in Mitosomes

Typically, enzymes responsible for sulfate activation are localized in the cytosol and/or plastids. Cytosolic localization is seen in non-photosynthetic and some plastidial organisms, whereas localization in plastids is seen in photosynthetic organisms. However, two exceptions have been reported: One is the nuclear localization of a human APSK-AS bifunctional protein, PAPS synthase 1 [4,25]; the other is mitochondrial localization of enzymes responsible for PAPS production in *Euglena gracilis*, a photosynthetic protozoan [2,26,27].

*Entamoeba* sulfate activation is atypically localized in MROs [28,29]. MROs are generally possessed by anaerobic eukaryotes. The retained functions have diversified from those of canonical mitochondria to different degrees during the course of evolution [30,31,32]. MROs in *Entamoeba*, known as mitosomes, are among the most degenerated types [30,33].

The localization of sulfate activation in MROs was also found in *Mastigamoeba,* a free-living close relative of *Entamoeba* [34]. Nevertheless, unlike in *Entamoeba* (see Section 3.2), the biological role of sulfate activation in *Mastigamoeba* is unknown. The atypical localization of sulfate activation in MROs in these organisms raises intriguing questions: What is the biological benefit of this unique compartmentalization for these organisms? What molecule is responsible for trafficking sulfate, the initial substrate, from the external milieu through the cytosol to the matrix of MROs? Furthermore, in *Entamoeba*, PAPS, the product of sulfate activation, must be exported from the matrix of mitosomes to the cytosol because all SULTs, the enzyme catalyzing the sulfate transfer from PAPS to an acceptor, are localized in the cytosol in *E*. *histolytica* [28,35] (Figure 3). Therefore, what is the molecule responsible for PAPS trafficking? In *Entamoeba* mitosomes, this molecule was identified by a biochemical approach. A member of the mitochondrial carrier family was shown to act as a PAPS/ATP antiporter that imports ATP from, and exports PAPS to, the cytosol [35] (Figure 3). The molecule responsible for sulfate trafficking is probably a sodium/sulfate symporter. Five sodium/sulfate symporters are encoded in *Entamoeba* genomes (AmoebaDB) and at least one sodium/sulfate symporter is localized in mitosomes [28]. However, the determination of membrane localization and biochemical characterization of the five sodium/sulfate symporters is required.

### 3.2. Sulfolipids Are the Major Products Synthesized by Entamoeba SULTs

A SULT family was deduced to be present in *Entamoeba* from the available databases (AmoebaDB) but its functional characterization remains to be performed. The chromatography of metabolically-radiolabeled sulfated molecules showed a major fraction of sulfated molecules that were sulfolipids, and not sulfated proteins or water-soluble molecules [28]. However, a comprehensive analysis, for example using sulfated lipid-based lipidomics, needs to confirm that sulfolipids are exclusively synthesized by the sulfation pathway in *Entamoeba*. 

Among sulfolipids (SL-I-VII) detected in *E*. *histolytica*, SL-I was identified as cholesteryl sulfate and SL-IV and -V were a mixture of C_16–20_ fatty alcohol disulfates [11,36]. It is worth mentioning that fatty alcohol disulfates are previously unrecognized natural compounds [36]. The sulfolipids (SL-I-VII) that have so far been detected in *E*. *histolytica* were determined to be related to EhSULTs (EhSULT1-10) by a combination of single and multiple gene knockdown (Box 2) [11,36] (see Figure 3). SL-I (cholesteryl sulfate) synthesis is mediated by EhSULT6 [11]. SL-II-IV, the structures of which have not been identified, are synthesized by EhSULT1-5 and -7-9. SL-V and -VI (fatty alcohol disulfates) are synthesized by EhSULT1, -3-5 and -7-9. SL-VII, the structure of which has not been identified, is synthesized by EhSULT10. EhSULT1-5 and -7-9 are functionally redundant [36].

Box 2 **Multiple Gene Knockdown.**

*Entamoeba*, like humans and plants, has SULT and SF gene families. Therefore, a single gene knockdown does not always produce a phenotype. To overcome this limitation, a multiple gene knockdown system in *E*. *histolytica* [36] was developed, which is based on a standard single gene knockdown system [29,37]. It should be mentioned that this new genetic system can reproducibly knockdown up to six genes using a single vector [36].

Moreover, gene knockdown in *E*. *histolytica* trophozoites that decreased the synthesis of fatty alcohol disulfates retarded growth, while the knockdown of genes that significantly decreased cholesteryl sulfate synthesis produced no distinct phenotype, indicating cholesteryl sulfate to be dispensable in trophozoites. Eventually, cholesteryl sulfate was demonstrated to play an important role in encystation of *Entamoeba*. The addition of cholesteryl sulfate to in vitro cultured *Entamoeba invadens* dose-dependently elevated the number of cysts formed. Conversely, the dose-dependent impairment of cyst formation was observed when cholesteryl sulfate synthesis in *E*. *invadens* was reduced by treatment with different concentrations of chlorate, an inhibitor for AS [11]. Note that the in vitro culture of *E*. *invadens*, a reptilian parasite, has been adopted as a model system for the study of *Entamoeba* encystation [10,38]. *Entamoeba* can survive severe environmental changes, e.g., exposure to high acidic conditions of the human stomach, and nutrient deprivation and dehydration outside the host, by alternating its form between proliferative trophozoites and dormant cysts (see Figure 2). Therefore, *Entamoeba* utilizes sulfate metabolism to synthesize appropriate sulfolipids for the maintenance of the parasitic lifestyle, i.e., fatty alcohol disulfates for trophozoite proliferation and cholesteryl sulfate for cyst formation (Figure 3). Furthermore, like fatty alcohol disulfates and cholesteryl sulfate, the structural identification and functional characterization of the other terminal metabolites, SL-II-IV and -VII, can provide new insights for chemical biology as well as parasitology.

### 3.3. Two Previously Unrecognized Entamoeba Enzymes Responsible for Fatty Alcohol Disulfate Synthesis

*Entamoeba* relies on the host for its supply of cholesterol and fatty acids because *Entamoeba* does not possess these de novo synthetic pathways [39]. ^14^C-stearic acid was incorporated into radiolabeled bands corresponding to fatty alcohol disulfates [36], indicating that *E*. *histolytica* synthesizes fatty alcohol disulfates from environmental fatty acids. To complete the synthesis of fatty alcohol disulfates, at least two distinct enzymes are required that catalyze fatty alcohol production and hydroxylation at the omega position of the fatty acid, fatty alcohol and/or fatty alcohol sulfate (Figure 3). The former reaction forms fatty alcohol from fatty acid, which is usually mediated by fatty acyl-CoA reductase [40]. The latter reaction is mediated by cytochrome P450 family member proteins in animals and plants [41,42]. However, neither enzyme is encoded in *Entamoeba* genomes ([40]; AmoebaDB). Hence, the identification and characterization of the enzymes involved in fatty alcohol disulfate synthesis represents new avenues in enzymology.

### 3.4. SF

The AmoebaDB database shows the presence of an SF family in *Entamoeba*, but it has not been characterized. The evidence provided by the different types of analysis indicates several features of *Entamoeba* SFs [36]. *E*. *histolytica* possesses five SFs (EhSF1-5), all of which are atypically classified as Zn-dependent alkylSFs, and not the more frequent formylglycine-dependent SFs. A combination of phylogenetic, bioinformatic and cell biological analyses indicates that all five EhSFs are localized in the *E*. *histolytica* cytosol. These five EhSFs are suggested to be responsible for the degradation of SL-II-IV in *E*. *histolytica* by metabolic labeling in gene knockdown strains (Box 2) (Figure 3).

The evidence described in this Section (Section 3.1, Section 3.2, Section 3.3 and Section 3.4) for *Entamoeba* sulfated molecule synthesis and degradation, and the terminal metabolites and their physiological roles is provided using *E*. *histolytica* and *E*. *invadens*. As compared to *E*. *histolytica* and *E*. *invadens* studies, the biochemical and cell biological evidence is very limited in the study for *Entamoeba dispar*. However, *E*. *dispar* possesses the genes encoding three enzymes crucial for sulfate activation, AS, APSK, and IPP, and SULT and SF families ([11,36]; AmoebaDB). *E*. *dispar* is morphologically identical to *E*. *histolytica* and parasitizes human intestines but is avirulent [43]. Taken together, *Entamoeba* sulfated biomolecule synthesis and degradation cooperatively function as sulfolipid metabolism, which is necessary for the organism’s parasitism (see Section 3.1, Section 3.2, Section 3.3 and Section 3.4).

### 3.5. Lateral Gene Transfer (LGT) Acquisition of Unique Sulfolipid Metabolism in Entamoeba

**Figure 4:**
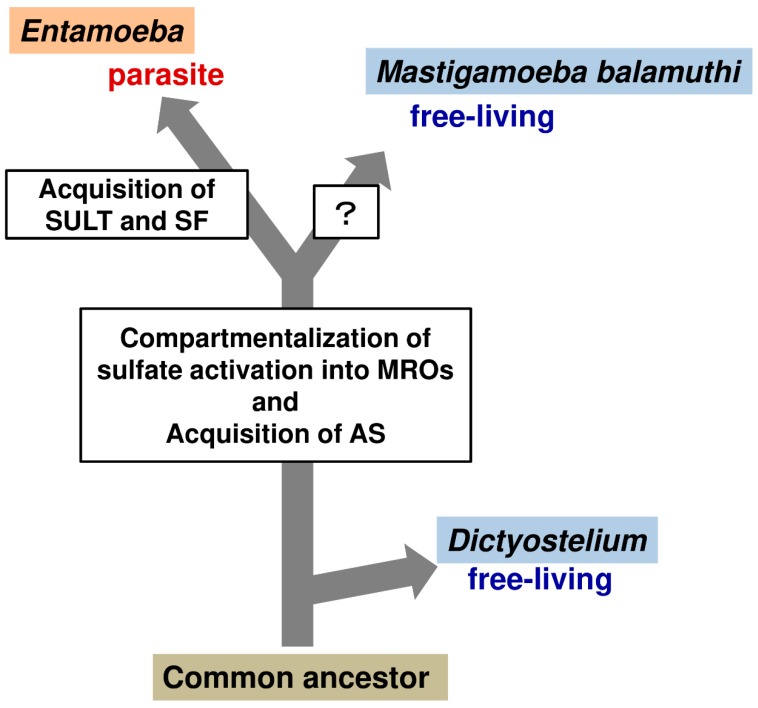
A plausible scenario for Amoebozoan lineage evolution. Schematic illustration of the evolutionary relationship among *Entamoeba*, *Mastigamoeba*, and *Dictyostelium* is drawn based on evidence described in this review.

The compartmentalization of *Entamoeba* sulfate activation in mitosomes is unique and consists of AS, a nonfunctional AS-APSK fusion protein, and IPP [28,29]. Furthermore, another unique feature of *Entamoeba* sulfate activation is that the gene encoding AS was transferred into *Entamoeba* via lateral gene transfer (LGT). Interestingly, *Mastigamoeba*, a non-parasitic close relative of *Entamoeba*, also has similar unique sulfate activation features of LGT acquisition [34] and domain structures (AS and a nonfunctional AS-APSK fusion protein), as well as the compartmentalization in MROs. The evidence for LGT acquisition was provided by several studies [1,28,34,44]. Phylogenetic trees for AS from different groups consistently showed that *Entamoeba* AS is more closely related to ASs in δ-proteobacteria than to those in other eukaryotes [28,34], and that *Entamoeba* AS clustered with *Mastigamoeba* AS [34]. These findings indicate that LGT acquisition of AS occurred in a common ancestor of *Entamoeba* and *Mastigamoeba*. The phylogenetic relationship of IPP, an enzyme functionally linked to sulfate activation [4], was also shown [34]. *Entamoeba* IPP clustered with *Mastigamoeba* IPP-1, both of which are localized in MROs [28,34], and this cluster located within the clade for eukaryotic IPPs. These findings indicate that all eukaryotic IPPs, including those of *Entamoeba* and *Mastigamoeba*, are diversified from a common ancestral IPP. In contrast to the phylogenetic trees for AS and IPP, those for APSK inferred by separate groups were inconclusive [28,34]. Therefore, its evolutionary history cannot be predicted. Nevertheless, similarity was found between *Entamoeba* and *Mastigamoeba* for a nonfunctional AS-like domain fused to a catalytic APSK domain in this order. A similar domain structure that retains a bifunctional AS-APSK protein was also present in *Dictyostelium discoideum*, a cellular slime mold belonging to the phylum Amoebozoa, [1], indicating that the gene encoding an AS-APSK fusion protein could have been present in the common ancestor of the lineage [34]. The sulfate activation is compartmentalized in MROs in both *Entamoeba* and *Mastigamoeba* [11,28,34]. Therefore, it is also important to unravel the evolutionary advantages of sulfate activation compartmentalization in MROs for these two lineages after a common ancestor of *Entamoeba* and *Mastigamoeba* acquired the gene encoding AS by LGT (see Figure 4).

Unlike protozoan parasites belonging to the genus *Entamoeba*, those in the genera *Plasmodium*, *Trypanosoma*, *Leishmania*, *Giardia*, and *Trichomonas*, but not *Toxoplasma*, do not possess the genes for sulfate activation [1]. A possible explanation for this finding is that these parasites may be able to salvage sulfated biomolecules from the host because similar to these parasites, there are several symbiotic genera, including *Encephalitozoon*, *Mycoplasma*, and *Symbiobacterium,* that lack sulfate activation [1].

Notably, the novel *Entamoeba* hallmark of sulfolipid metabolism, is not conserved in *Mastigamoeba*, a non-parasitic close relative of *Entamoeba*. Sulfolipid synthesis could not be detected in *Mastigamoeba* by metabolic labeling using radiolabeled sulfate and SULT and SF are not encoded in the *Mastigamoeba* genome ([11], NCBI). Furthermore, phylogenetic trees independently inferred by different groups show that LGT acquisition of SULTs and SFs occurred only in the *Entamoeba* lineage [11,36,44,45], while the acquisition of AS took place in a common ancestor of *Entamoeba* and *Mastigamoeba* [1,11,34]. The origin of *Entamoeba* SFs is demonstrated to be an ancestor of *Bacteroides* sp., which belongs to a different Proteobacteria phylum, although the origin of *Entamoeba* SULTs remains unknown because of low resolution of the inferred phylogenetic tree [11,36,44,45]. *Bacteroides* sp. is a commensal microorganism of the human gut microbiota [46], and shares a habitat with *E*. *histolytica*, a parasitic microorganism. Hence, these findings indicate that after branching from the common *Entamoeba* and *Mastigamoeba* lineage, LGT acquisition of catabolic SF and anabolic SULT enzymes occurred in an ancestor of *Entamoeba*, which promoted sulfur metabolism to evolve as sulfolipid metabolism, enabling the ancestor to thrive as a parasitic lineage, *Entamoeba* (Figure 4). It is worth mentioning that these findings provide evidence to substantiate LGT, which is a process that generates new genes, and can drive adaptive evolution [47], contributing to the establishment of a parasitic lineage by conferring a novel hallmark in ubiquitous sulfur metabolism.

## 4. Pharmaceutical Exploitation of *Entamoeba* Sulfolipid Metabolism

As described in Section 3.1, Section 3.2, Section 3.3 and Section 3.4, *Entamoeba* sulfolipid metabolism has pleiotropic roles in the maintenance of the life cycle, which consists of trophozoite and cyst stages. The capabilities of *E*. *histolytica* trophozoites and cysts are clinically linked to amoebiasis [8,9]. *E*. *histolytica* sulfolipid metabolism is, therefore, an ideal target for developing new preventive measures against amoebiasis, such as anti-amoebic and amoebiasis transmission-blocking drugs. Among enzymes directly involved in the synthesis and degradation of sulfolipids, *E*. *histolytica* AS and APSK (EhAS and EhAPSK) are more suitable targets than EhSULT1-10 and EhSF1-5 because the two enzymes fulfill the requirements of a target for drug development. They are the sole enzymes that function at an early step in the pertinent metabolism and do not show functional redundancy.

A recent study focusing on *E*. *histolytica* sulfolipid metabolism presented distinct lines of evidence that *E*. *histolytica* APSK (EhAPSK), the enzyme catalyzing the latter step of sulfate activation, is a rational target for amoebiasis therapy. Further, 2-(3-fluorophenoxy)-N-[4-(2-pyridyl)thiazol-2-yl]-acetamide (A-D-11), 3-phenyl-N-[4-(2-pyridyl)thiazol-2-yl]-imidazole-4-carboxamide (A-H-11), and auranofin, are leads for developing new drugs against amoebiasis [48]. An EhAPSK-based combination approach of an in silico molecular docking analysis and an in vitro enzyme activity assay enabled the screening of 400 chemicals in the Pathogen Box of the Medicines for Malaria Venture (MMV; https://www.pathogenbox.org/), from which 15 compounds were identified that inhibit EhAPSK activity. Among them, the above three compounds dose-dependently impaired sulfolipid synthesis in live *Entamoeba* cells, resulting in the inhibited trophozoite proliferation and cyst formation. These results indicate that A-D-11, A-H-11, and auranofin all halt biological processes essential to the *Entamoeba* life cycle by targeting EhAPSK, which impairs the synthesis of sulfolipids, including fatty alcohol disulfates and cholesteryl sulfate (Figure 3). Furthermore, A-D-11 and A-H-11 showed almost no cytotoxic activity against a human cell line, indicating that they are promising leads for the development of new drugs against amoebiasis. In contrast, auranofin showed an adverse effect on human cells in vitro [48,49]. Nevertheless, auranofin was approved as an orally administered compound for the treatment of rheumatoid arthritis by the FDA in 1985, and was shown to be effective in vitro and in vivo against *E*. *histolytica* [50]. Furthermore, phase I clinical trial results of auranofin support auranofin safety and provide important pharmacokinetics data that support its potential use as an anti-amoebic drug [51].

The evidence, which is apparently paradoxical to the above EhAPSK-based study [48], has been reported, indicating that auranofin targets thioredoxin reductase (TrxR) in *E*. *histolytica* (EhTrxR) [50]. Consistently, EhTrxR was inhibited by auranofin during the turnover of Trx in an in vitro assay using a purified recombinant enzyme. However, the x-ray structure analysis was not available for either the binding of auranofin to cysteine thiol groups in the catalytic C(X)_2_C motif or to a substrate (Trx)-binding site of EhTrxR [52]. The authors, therefore, hypothesize that auranofin targets two distinct enzymes in *Entamoeba*: APSK and TrxR, which explains this apparent discrepancy [48].

## 5. Concluding Remarks and Future Perspectives

Sulfur metabolism is ubiquitous among and crucial for living organisms. Organisms usually synthesize sulfated biomolecules from sulfate available in their habitats via sulfur metabolism, and some organisms degrade sulfur-containing products. *Entamoeba* synthesizes and degrades sulfolipids, indicating that the biosynthesis and degradation pathways uniquely constitute sulfolipid metabolism. Importantly, cholesteryl sulfate and fatty alcohol disulfates, the terminal metabolites, play separate, important roles in the *Entamoeba* life cycle. The remaining synthesized sulfolipids, the structures of which have not been determined, also contribute to the parasitic lifestyle. Ultimately, the molecular mechanisms by which the sulfolipids synthesized participate in *Entamoeba* physiology need to be uncovered.

As well as the terminal products, *Entamoeba* sulfolipid metabolism shows unique features, as described in this review. These raise important and intriguing questions: What is the biological and evolutionary benefit for *Entamoeba* to limit the terminal metabolites to sulfolipids? What is the evolutionary advantage for both parasitic *Entamoeba* and free-living *Mastigamoeba* to compartmentalize sulfate activation into MROs? What are the enzymes involved in the synthesis of fatty alcohol disulfates? Answering these issues will not only give new insights in sulfur metabolism in conjunction with other lipid metabolism, but also open new paradigms linked to chemical and evolutionary mitochondrial biology, and biochemistry.

*Entamoeba* sulfolipid metabolism is also important as a therapeutic subject. EhAPSK, an enzyme that only functions in the early steps in *Entamoeba* sulfolipid metabolism, is a rational target for amoebiasis therapy. Meanwhile, from a chemical biology aspect, searching for inhibitors of AS, SULTs, SFs and APSK potentially provides useful inhibitors to investigate *Entamoeba* physiology because they exert specific or pleiotropic roles in the *Entamoeba* life cycle. However, some enzymes show functional redundancy, which hampers the search for inhibitors. The inhibitors obtained may not only be advantageous for the basic study of *Entamoeba*, in which a gene knockout system has not been developed, but may also be leads for developing new anti-amoebic and amoebiasis transmission-blocking drugs, the combined administration of which can potentially lead to amoebiasis eradication.

In conclusion, sulfur metabolism is not a traditionally studied area of the metabolism. The study of *Entamoeba* sulfolipid metabolism can be broadened by linking it to other ever-expanding fields, such as mitochondrial evolution and the human gut microbiome.
